# 2317. Persistent Bacterial Carriage and Hospital Transmission Detected by Whole Genome Sequence Surveillance

**DOI:** 10.1093/ofid/ofac492.1898

**Published:** 2023-04-06

**Authors:** Alexander Sundermann, Marissa Griffith, Kady Waggle, Graham Snyder, Daria Van Tyne, Jane Marsh, Lee Harrison

**Affiliations:** University of Pittsburgh, Pittsburgh, Pennsylvania; University of Pittsburgh, Pittsburgh, Pennsylvania; University of Pittsburgh, Pittsburgh, Pennsylvania; University of Pittsburgh, Pittsburgh, Pennsylvania; University of Pittsburgh, Pittsburgh, Pennsylvania; University of Pittsburgh, Pittsburgh, Pennsylvania; University of Pittsburgh Medical Center, Pittsburgh, Pennsylvania

## Abstract

**Background:**

Healthcare-associated infections can be acquired via transmission of pathogens within the healthcare setting. Often, patients are assumed to have short duration (< 90 days) of transmissibility with bacterial pathogens after developing a clinical infection. This assumption may wrongly exclude patients as sources of transmission when they have persistent bacterial carriage. We studied patients with persistent carriage and associated transmission using whole genome sequencing surveillance.

**Methods:**

Patient culture positive isolates for select bacterial pathogens between 11/2016 and 11/2019 were collected if the patient was housed in the hospital for ≥3 days or had a recent healthcare exposure in the prior 30 days. Isolates were considered genetically related with ≤15 SNPs for all organisms except *C. difficile* (≤2 SNPs). Patients with serial isolates separated by >100 days were examined for other patients with related isolates and epidemiological commonalities between the first and last culture dates.

**Results:**

There were 779 related isolates from 369 unique patients (range 2-11 isolates/patient). The mean time from first to last culture date was 81.9 days (median 33 days, range 1-899 days) (Figure 1). 77 patients had isolates that were related to another patient of which 18 (23%) patients had >100 days between their first and last isolate (median 216, range 103-899). Of these, 9 (50%) patients had epidemiological links with another patient between their first and last isolate culture dates. The median time from exposure to positive culture date of the exposed patient was 14 days (mean 34, range 2-115). An example of potential transmission is shown in Figure 2.

Days between the first and last related isolates within the same patient

Example of patient with persistent carriage of K. pneumoniae and evidence of transmission to another patient

**Conclusion:**

Some patients had persistent carriage with the same strain for over two years and appear to be a potential source of ongoing transmission to other patients. WGS surveillance, in addition to detecting outbreaks, can identify patients with persistent colonization as potential a transmission source. Healthcare outbreak investigations should include patients with persistent carriage as potential sources based upon temporal restrictions.

**Disclosures:**

**Graham Snyder, MD, SM**, Infectious Diseases Connect: Advisor/Consultant **Daria Van Tyne, PhD**, Century Therapeutics, Inc: Advisor/Consultant

